# Path Planning of Mobile Robots Based on a Multi-Population Migration Genetic Algorithm

**DOI:** 10.3390/s20205873

**Published:** 2020-10-17

**Authors:** Kun Hao, Jiale Zhao, Kaicheng Yu, Cheng Li, Chuanqi Wang

**Affiliations:** 1 School of Computer and Information Engineering, Tianjin Chengjian University, Tianjin 300384, China; 18530185645@163.com (J.Z.); licheng@mun.ca (C.L.); 2 School of International Education, Tianjin Chengjian University, Tianjin 300384, China; kaichengyu@tcu.edu.cn; 3 Tianjin Keyvia Electric Co., Ltd, Tianjin 300384, China; wangcq@keyvia.cn

**Keywords:** genetic algorithm, path planning, multi-population, migration mechanism, mobile robot

## Abstract

In the field of robot path planning, aiming at the problems of the standard genetic algorithm, such as premature maturity, low convergence path quality, poor population diversity, and difficulty in breaking the local optimal solution, this paper proposes a multi-population migration genetic algorithm. The multi-population migration genetic algorithm randomly divides a large population into several small with an identical population number. The migration mechanism among the populations is used to replace the screening mechanism of the selection operator. Operations such as the crossover operator and the mutation operator also are improved. Simulation results show that the multi-population migration genetic algorithm (MPMGA) is not only suitable for simulation maps of various scales and various obstacle distributions, but also has superior performance and effectively solves the problems of the standard genetic algorithm.

## 1. Introduction

With the development of society, mobile robots are playing an increasingly important role in modern life. Mobile robots can autonomously move and operate according to different assigned tasks and have been widely used in the military, medical, manufacturing, entertainment, logistics and other fields [1,2,3]. The path planning problem is a hot topic in the field of robotics research. It requires robots to find an optimal or suboptimal path from the starting position to the target position according to some specific performance index (such as distance, time, etc) in a working environment with obstacles [4,5].

Path planning problems are generally divided into global path planning and local path planning. In global path planning, a path search is carried out in a known environment. On the contrary, local path planning is relatively complex because the environment may be partially or completely unknown. According to the information of obstacles, the working environment of the robot can be divided into the fully known environment, partly known environment, completely unknown environment and dynamic environment [6]. The quality of robot path planning can be evaluated according to path length, path smoothness, energy consumption or risk degree [7].

According to the different stages of the path planning algorithm development, the algorithms can be divided into two categories: fast-exploring random tree method [8], artificial potential field method [9], the visible method [10], A* algorithm [11] as representative traditional algorithms. Intelligent algorithms represented by genetic algorithm [12], ant colony algorithm [13], particle swarm algorithm [14], immune cloning algorithm [15]. In [8], Janson theoretically proved that the use of deterministic low-dispersion sampling plan usually makes the RRT algorithm display superior performance. In [9], Yu proposed an improved artificial potential field method. This method uses the strength of the potential field instead of the force vector to plan the path of the mobile robot. This method can better realize the path planning of the mobile robot in a dynamic environment. In [10], Blasi first proposed a real-time collision avoidance algorithm based on visibility graph method. The algorithm solves the optimization problem through a piecewise linear path with the smallest cost. In [11], Le proposed an improved A* algorithm. The algorithm can automatically generate path points to improve the path coverage of the robot. These traditional path planning algorithms are light and small, with less calculation and easy to understand. Such algorithms are often used for small-scale maps with fewer obstacles. However, these traditional path planning algorithms are not suitable for large-scale maps or maps with many obstacles, and it is easy to fall into a locally optimal solution. In [12], Park proposed a new multi-population genetic algorithm. The algorithm can solve complex communication problems and multi-label feature selection problems. In [13], Beschi used an ant colony algorithm to solve complex motion planning problems after discretizing the task space. In [14], Strąk proposed a discrete particle swarm optimization algorithm to solve the dynamic traveling salesman problem. The algorithm can automatically set the parameter values of the discrete particle swarm optimization algorithm. Simulation results show that the algorithm is suitable for large-scale dynamic traveling salesman problems. Aiming at the multi-objective optimization problem, reference [15] proposed an immune cloning algorithm based on the reference direction method. The algorithm uses the reference direction method to guide the selection and cloning of active populations. The simulation results show that the algorithm still has strong competitiveness in complex environments. Compared with traditional algorithms, these intelligent algorithms are suitable for both small-scale maps and large-scale maps. They can be used for both global path planning and local path planning, and can effectively avoid local optimal solution problems.

This article uses the genetic algorithm to study path planning. The genetic algorithm is designed according to the evolutionary laws of organisms in Nature. This algorithm is a randomized search method that simulates natural selection theory and biological genetic mechanisms. Because the genetic algorithm has the advantages of strong robustness and parallelism, it is widely used in path planning. Forrest [16] summarized the standard genetic algorithm (SGA) proposed by Holland and pointed out that SGA has the advantages of flexible search and strong scalability. However, SGA also has disadvantages such as low quality of convergent individuals, many iterations required for convergence, easy to fall into local optimal solutions, and poor population diversity. Hu Jun et al. [17] initialized the population by introducing chaotic sequences and a heuristic method based on environmental knowledge to improve the quality of the initial population. However, this method is very slow at generating individuals in a multi-obstacle environment and may even fail to produce effective individuals. Shi et al. [18] proposed a new coding method based on projecting two-dimensional data to one-dimensional data. This coding method can reduce the computational complexity of the algorithm model. However, this coding method can only generate fixed-length codes. This will affect the quality of the generated path. This encoding method is also not suitable for large-scale maps. Guo et al. [19] proposed an improved genetic algorithm (IGA) by improving the crossover mutation operator of SGA. Compared with SGA, IGA can converge faster. However, the quality of convergent individuals is not good, and the shortcomings of poor population diversity are still not significantly improved. Based on the apoptosis theory proposed by Yigong, Zhang et al. [20] improved the selection operator of SGA. Then they proposed the programmed cell death evolutionary algorithm (PCDA). Compared with SGA, PCDA has improved performance indexes such as program running time, iterations required for convergence, individual quality of convergence, and population diversity. However, there is also the problem that population diversity drops sharply in the middle and late iteration.

Through the above analysis, this paper proposes a path planning method based on the multi-population migration genetic algorithm (MPMGA). This paper makes the following innovations and contributions: (1) A new algorithm framework is proposed to enhance the parallelism of the algorithm. (2) Compared with other algorithms, MPMGA can be used for both global static path planning and local dynamic path planning and MPMGA shows good performance in both. (3) Compared with other algorithms, MPMGA has better feasibility and effectiveness in actual maps and good applicability in simulation maps. (4) No matter what the scale of the map and no matter how the obstacles are distributed, MPMGA can always generate high-quality and effective planning paths. (5) MPMGA proposes migration operators and optimization operators. The migration operator can speed up the algorithm convergence speed and enhance the diversity of the population. Optimization operators can further improve the quality of convergent individuals. (6) MPMGA improves the population initialization process, crossover operator and mutation operator. MPMGA improves the quality of the initial population by improving the population initialization process. MPMGA further enhances the global search ability of the algorithm by improving the crossover operator. MPMGA further enhances the local search ability of the algorithm by improving the mutation operator.

The remainder of this paper is organized as follows: Section 2 introduces the algorithm framework of MPMGA. Section 3 introduces the modeling method of the two-dimensional space environment and the preprocessing process for irregular obstacles. In Section 4, we introduce the various aspects of MPMGA in detail, including encoding methods, population initialization process, cross mutation operator, migration operator, etc. In Section 5, we compare the performance of each algorithm and the quality of path planning of each algorithm in two different scale simulation maps through simulation programs. We analyze in detail the reasons why each algorithm produces good or poor performance. In Section 6, we summarize the entire paper and discuss the applicability and problems of MPMGA on a real mobile robot. According to these issues and limitations, we discuss future work.

## 2. MPMGA Framework

We propose MPMGA based on the standard genetic algorithm. MPMGA randomly divides a large population into several small populations with identical numbers. By assigning different functions to different small populations, MPMGA successfully makes the high-quality individuals in ordinary populations rise to high-quality populations and inferior individuals in high-quality populations sink to ordinary populations and randomly exchanges individuals from different populations.

**Figure 1:**
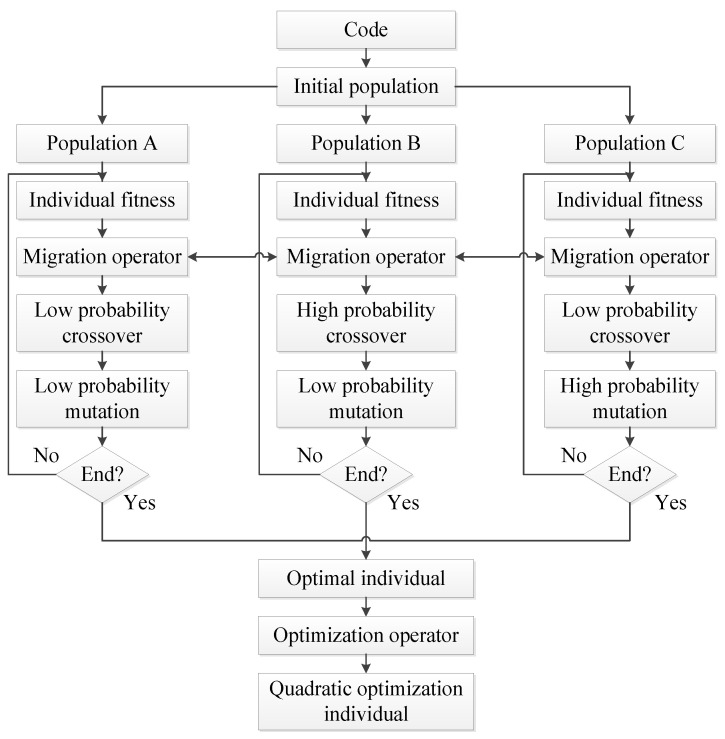
MPMGA frame diagram.

As shown in Figure 1, the initial population is randomly divided into populations A–C. After evaluating the fitness of different individuals, according to the fitness, we can complete the migration operations such as the rising, sinking and communication of individuals among different populations. Then we perform the crossover and mutation operations. After the evolution is over, the best individual can be obtained. Through performing the second optimization on the best individual, the quadratic optimization individual is obtained.

## 3. Environment Modeling

**Figure 2:**
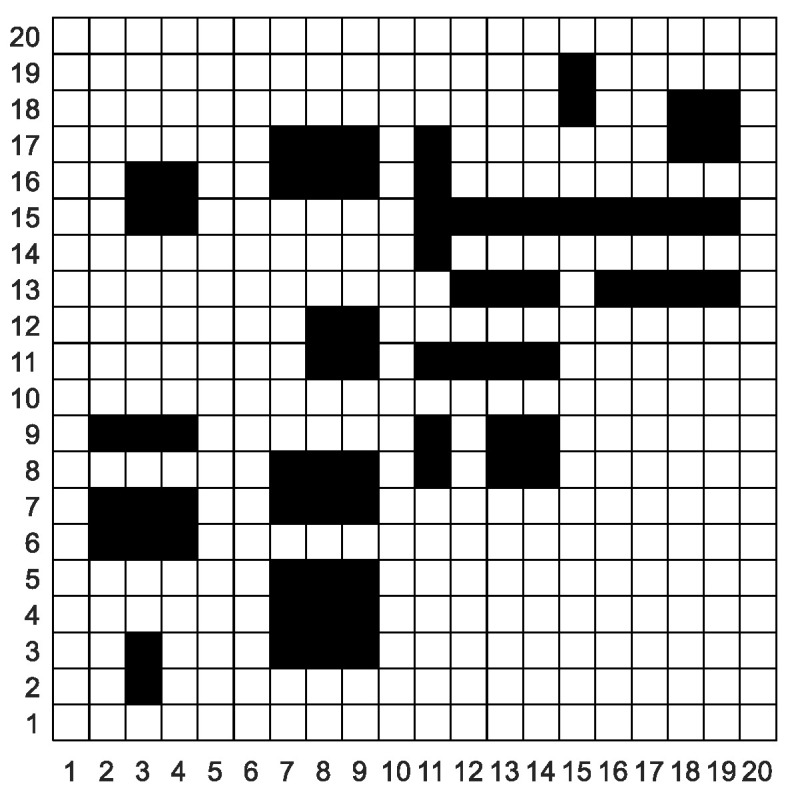
Grid map.

In this paper, the grid method is used to build the environment model. The grid method is a method that divides the two-dimensional workspace of mobile robots into several grids of the same size. As shown in Figure 2, the entire two-dimensional workspace is divided into a 20 × 20 grid map by using the grid method. In the grid map, the numbers are 0, 1, 2, 3, 4...399 from left to right and bottom to top. The white grid represents the feasible area, and the black grid represents the infeasible area, i.e., the obstacle area. Grid coordinates are represented by grid center points.

The corresponding relationship between grid number and grid coordinate is

(1) $$\left\{ \begin{array}{l} x=\mathrm{mod}\left(p,N\right)+1\\ y=fix\left(p/N\right)+1 \end{array}\right.$$

(2) p=(x−1)+(y−1)∗N

Equation (1) converts grid numbers into grid coordinates and Equation (2) converts grid coordinates into grid numbers. In the Equations (1) and (2), *p* is the grid number, *(x,y)* is the coordinate point corresponding to the grid, *N* is the grid number per row, mod is the remainder operation, and *fix* is the rounding operation.

To improve the security of the grid map and the efficiency of the algorithm, the following preprocessing is required:

(1) The mobile robot is equivalent to the mass point, and the obstacle is expanded. The expansion size is the sum of the radius and reserved safety distance of the mobile robot.
(2) If the obstacle is irregular, the grid is marked in black, where the obstacle is located.
(3) If all eight directions of a white grid are black grids, this white grid is also marked as a black grid.

## 4. Algorithm Design

### 4.1. Coding Mode

**Figure 3:**
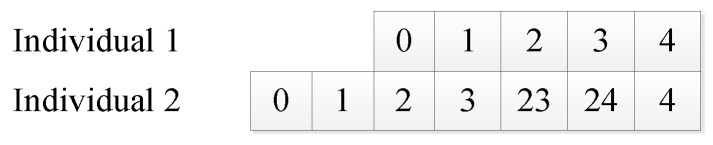
Variable-length real-number coding.

Common coding methods include binary coding, gray coding, floating-point coding, real coding, permutation coding, etc. In this paper, variable-length real-number coding is used. Variable-length real-number coding refers to real-number coding with a variable chromosome length (as shown in Figure 3).

### 4.2. Initial Population

**Figure 4:**
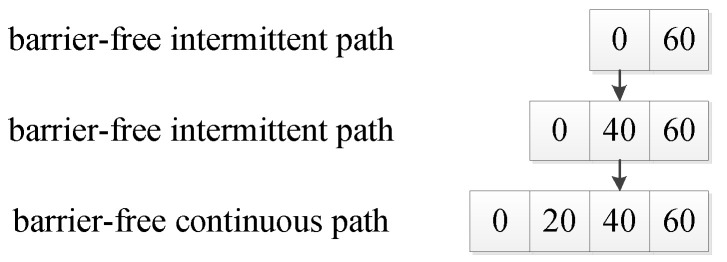
The connection operator.

The barrier-free intermittent path refers to a path that selects a series of free grids between the starting point and the target point and does not require continuity between the grids [21]. Based on the barrier-free intermittent path, this paper connects the barrier-free intermittent path into a barrier-free continuous path through the connection operator. Then we use the deletion operator to delete the circular partial path in the barrier-free continuous path; thus, we can generate high-quality initial populations. The connection operator refers to an operator that connects a barrier-free intermittent path into a barrier-free continuous path (as shown in Figure 4). For example 0,60 is a barrier-free intermittent path. It becomes a barrier-free continuous path such as 0, 20, 40, 60, after the operation of the connection operator. The connection method is the intermediate value insertion method. i.e., if two adjacent path points in a path are not continuous, the middle grid of the connection line between two path points is inserted into the middle of two path points. If the inserted grid is an obstacle grid, we replace the obstacle grid with a free grid around the obstacle grid. Then we repeat the insertion process in such a loop until the entire path becomes a barrier-free continuous path.

**Figure 5:**
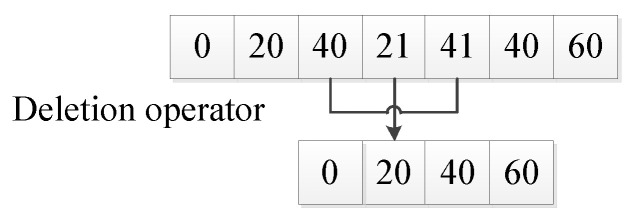
The deletion operator.

The deletion operator refers to an operator that deletes a circular partial path in a barrier-free continuous path. For example: in paths 0, 20, 40, 21, 41, 40, 60, paths 21, 41, 40 are circular partial paths; then, the path after using the deletion operator is: 0, 20, 40, 60.The deletion operator deletes the circular partial path by removing any repeated path point and the partial path between repeated path points in the barrier-free continuous path (as shown in Figure 5).

**Figure 6:**
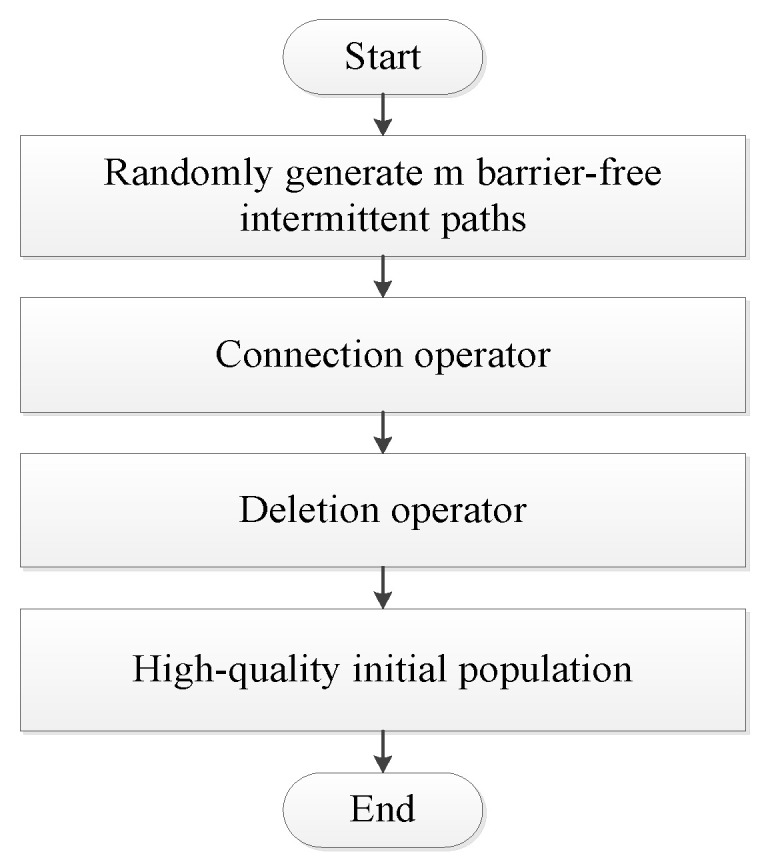
Flow chart of the initial population generation.

The generation process of the initial population is shown in Figure 6.

### 4.3. Fitness Function

Fitness is often used to evaluate the quality of path individuals. High fitness implies good individual quality. Compared with the traditional single-objective optimization, this paper comprehensively considers two factors: path length and path smoothness. In the form of a weighted sum, the multi-objective optimization problem is transformed into a single-objective optimization problem. The fitness function is defined as follows:

(3) fitness=α∗f1+β∗f2

(4) α+β=1

where *fitness* is the total fitness function, *f1* is the fitness function of the path length, *f2* is the fitness function of the path smoothness, and *α* and *β* are the weights of the two fitness functions.

*f1* and *f2* are defined as follows:

(5) $$\left\{ \begin{array}{l} f1=1/path\\ f2=c/smoothness \end{array}\right.$$

where *path* is the length of the path, *smoothness* is the smoothness of the path and *c* is a precision coefficient and *c* is a fixed constant. By adjusting *c*, *f2* and *f1* can be controlled to maintain the same order of magnitude.

Assuming that a specific route is composed of n waypoints, the coordinate of the i-th waypoint is *P_i_(x_i_,y_i_)* and the coordinate of the i + 1 th waypoint is *P_i+1_(x_i+1_,y_i+1_)*; then, the path length can be expressed as:

(6) $$path=\sum_{i=1}^{n-1}\sqrt{{\left(x_{i+1}-x_{i}\right)}^{2}+{\left(y_{i+1}-y_{i}\right)}^{2}}$$

**Figure 7:**
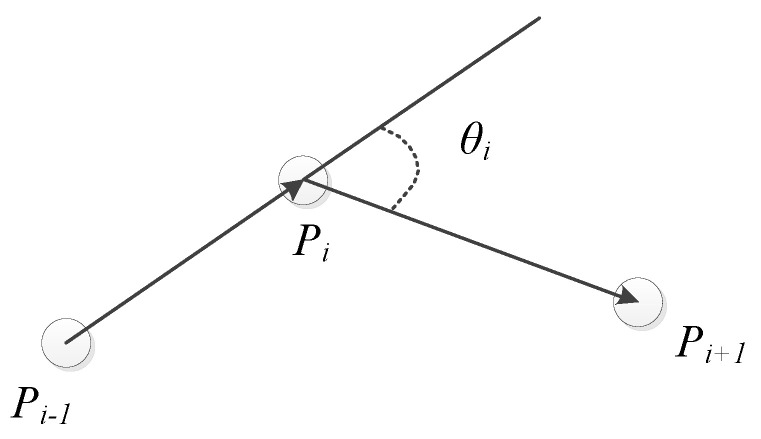
Schematic diagram of the rotation angle between path segments.

Suppose that there are three continuous path points *P_i−1_, P_i_, P_i + 1_*, and two path segments among the three path points: *P_i−1_P_i_, P_i_P_i + 1_*. Let *θ_i_* be the rotation angle between the two path segments and *α_i_* be the included angle between the two path segments, i.e., *π−θ_i_*, as shown in Figure 7.

There are:

(7) $$k1=\left\{ \begin{array}{l} \left(y_{i}-y_{i-1}\right)/\left(x_{i}-x_{i-1}\right),x_{i}\neq x_{i-1}\\ \infty ,x_{i}=x_{i-1} \end{array}\right.$$

(8) $$k2=\left\{ \begin{array}{l} \left(y_{i+1}-y_{i}\right)/\left(x_{i+1}-x_{i}\right),x_{i+1}\neq x_{i}\\ \infty ,x_{i+1}=x_{i} \end{array}\right.$$

(9) $$\theta_{i}=\left\{ \begin{array}{l} 0,k1=\infty \;k2=\infty \\ 0,k1=k2\;k1,k2\neq \infty \\ \pi -\alpha_{i},other \end{array}\right.$$

(10) $$\left\{ \begin{array}{l} a1={\left(x_{i-1}-x_{i}\right)}^{2}+{\left(y_{i-1}-y_{i}\right)}^{2}\\ b1={\left(x_{i+1}-x_{i}\right)}^{2}+{\left(y_{i+1}-y_{i}\right)}^{2}\\ c1={\left(x_{i-1}-x_{i+1}\right)}^{2}+{\left(y_{i-1}-y_{i+1}\right)}^{2}\\ a=\sqrt{a1}\\ b=\sqrt{b1}\\ c=\sqrt{c1} \end{array}\right.$$

(11) $$d_{i}=\left(a1+b1-c1\right)/\left(2*a*b\right)$$

(12) $$\alpha_{i}=a\cos\left(d_{i}\right)$$

where Equations (10)–(12) use the inverse cosine function to calculate angle *α_i_* between the two path segments. Equations (7)–(8) calculate the slope of the two path segments. Due to the inherent defects of the inverse cosine function, in Equation (9), the value of rotation angle *θ_i_* is discussed by using the relationship between the slopes of the two path segments.

Path smoothness is a penalty set according to the value of rotation angle *θ_i_*. The smoothness of the path can be measured by *smoothness* as follows:

(13) $${smoothness}_{i}=\left\{ \begin{array}{l} 0,\theta_{i}=0\\ 5,0<\theta_{i}<\pi /2\\ 25,\theta_{i}=\pi /2\\ 125,\theta_{i}>\pi /2 \end{array}\right.$$

(14) $$smoothness=\sum_{i=2}^{n-1}{smoothness}_{i}$$

In Equation (13), a larger the rotation angle *θ_i_* corresponds to a greater smoothness value of the path, which indicates that the path is not smooth. In addition, when the value of *θ_i_* is too large, the mobile robot has great challenges in terms of energy consumption and safety, so it must be given a high penalty.

### 4.4. Migration Operator

Common selection strategies include roulette selection, elite selection, tournament selection, truncation selection, etc. However, there are some problems in these selection strategies, such as serious homogenization, easy convergence to local optimal solutions and loss of population diversity. The most prominent problem is the serious homogenization phenomenon. The phenomenon of homogenization implies that as the number of iterations increases, a particular individual will appear in large numbers in the population. The phenomenon of homogeneity will make the effect of the crossover operator gradually decrease as the number of iterations increases. When the entire population is composed of the same individuals, the crossover operator is completely invalid.

**Figure 8:**
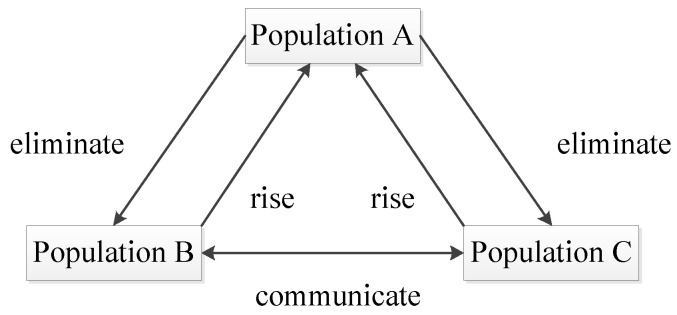
Migration process.

Based on the ideas of population mobility and social division of labor proposed in the reference [22,23,24], this paper proposes a migration operator. The migration operator refers to a comprehensive mechanism that assigns different functions to different small populations, maintains normal communication among different small populations and ensures that high-quality individuals in ordinary populations rise to high-quality populations and inferior individuals in high-quality populations sink to ordinary populations. The migration process of the migration operator is shown in Figure 8.

In Figure 8, three small populations are given different functions by setting different crossover rates and mutation rates. By giving population A low crossover rate and low mutation rate. We make population A well preserve high-quality individuals. This way can prevent the loss or destruction of high-quality individuals. Therefore, population A is designated as a high-quality population. By giving population B high crossover rate and low mutation rate and giving population C low crossover rate and high mutation rate. We make population B and population C act as resource banks. This way can provide many crossover and mutation individuals and is conducive to expanding the solution space search coverage. Hence, we designate population B and population C as ordinary populations.

In the migration process of Figure 8, several high-quality individuals in population B and population C rose to population A to be preserved, and a part of the poor individuals in population A were eliminated into population B and population C to act as raw materials for crossover or mutation. The latter process can play the role of waste utilization. In addition, a certain individual communication mechanism has been maintained between population B and population C to break the gap between the two populations.

In summary, the migration operator has the advantages of accelerating the convergence rate, increasing the population diversity, breaking the local optimal solution and solving the serious homogenization of the population individual in the middle and late iterations.

### 4.5. Crossover Operator

The crossover operator is an operator that generates new individuals through the crossover recombination of two individuals. Common crossover operations include single-point crossover, multipoint crossover, uniform crossover, etc. Reference [25] uses a single-point crossover method and notes that single-point crossover is more efficient and easier to implement than other crossover methods.

Single-point crossover randomly selects any path point with the same grid number in the two paths and exchanges the chromosome fragments after the selected path point to form two new individuals. This crossover method is also called homologous single-point crossover [26].

This paper proposes a heterologous single-point crossover based on the homologous single-point crossover and adopts the individual reception method proposed in the reference [27]. If the individual after crossover is better than the individual before crossover, then the individual after crossover is accepted. If the individual after crossover is worse than the individual before crossover, then the individual before crossover is accepted. The heterogeneous single-point crossover operator no longer looks for a common path point with identical grid numbers but finds a pair of path points that satisfy the condition that the two new paths are still continuous after the crossing. Such a group of path points must satisfy two conditions:

(1) The grid corresponding to the *i*-th path point in the first path and the grid corresponding to the *j* + 1-th path point in the second path are continuous.
(2) The grid corresponding to the *i* + 1-th path point in the first path and the grid corresponding to the *j*-th path point in the second path are continuous.

**Figure 9:**
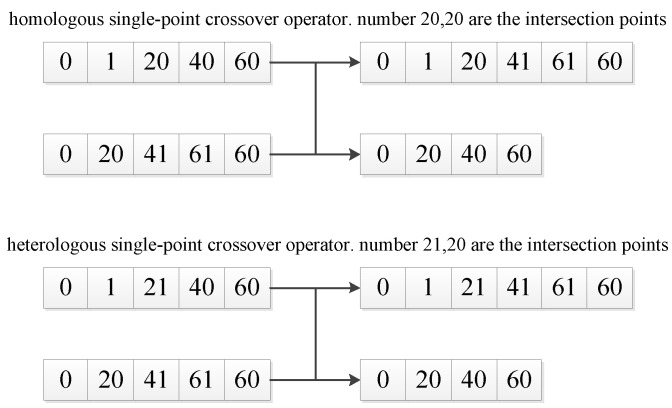
Crossover graph.

Such a set of path points *i, j* is the cross path point for which the heterogeneous single point cross operator is looking. The two new individuals obtained by crossing the chromosome segment after the *i*-th path point in the first path and the chromosome segment after the *j*-th path point in the second path are still unobstructed continuous paths (as shown in Figure 9).

The cross-path point searched by the heterologous single-point crossover operator includes the cross-path points searched by the homologous single-point crossover operator. So the homologous single-point crossover operator is a special case of the heterologous single-point crossover operator.

### 4.6. Mutation Operator

**Figure 10:**
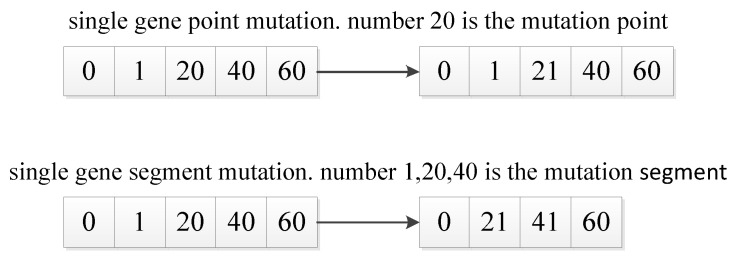
Mutation graph.

MPMGA proposes the single-gene segment mutation and uses a simulated annealing algorithm to improve the receiving method of mutant individuals. Single-gene segment mutation refers to the mutation way of mutating a specific gene segment rather than mutating a certain gene point (as shown in Figure 10). i.e., it mutates a random gene segment. Specific operation is to randomly delete a specific gene segment on the individual and then use the connection operator to repair the damaged individual. Single-gene segment mutation ensures the continuity and accessibility of the individual after the mutation. Single-gene segment mutation greatly improves the quality of the mutation and enhances the ability to explore the solution space.

Compared with the method of full reception or optimal reception, the simulated annealing algorithm is more flexible for receiving mutated individuals. When the mutant individual is better than the original individual, the mutant individual will be accepted. When the mutant individual is worse than the original individual, the mutant individual will be accepted with a certain probability. The advantage is that it can consider the retention of high-quality individuals and give some tolerance to inferior individuals to expand the diversity of the population.

Assume that the individual before mutation is represented as *old*, the individual after mutation is represented as *new*, and *fitness()* represents the fitness of an individual. Then, the simulated annealing algorithm receives the mutation individual probability equation as follows:

(15) a=(fitness(old)−fitness(new))/T

(16) $$p\left(new\right)=\left\{ \begin{array}{l} 1,fitness\left(new\right)>fitness\left(old\right)\\ 1/\left(1+e^{a}\right),fitness\left(new\right)\leq fitness\left(old\right) \end{array}\right.$$

(17) $$T=c_{1}*w^{t}$$

In Equation (15), *T* is the current temperature and *a* is a parameter. In equation (16), *p(new)* is the probability of receiving the mutated individual. In Equation (17), *c_1_* is the initial temperature, *w* is the temperature decay rate and *t* is the current number of iterations. Obviously, when the number of iterations *t* increases, temperature *T* will decrease and the tolerance for individuals who become worse after mutation will decrease. Until a certain generation, the algorithm will no longer tolerate individuals who become worse after mutation.

### 4.7. Optimization Operator

**Figure 11:**
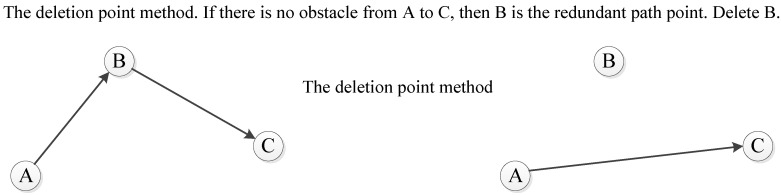
The deletion point method.

The initial population will converge to the optimal individual through several iterations. Then the optimization operator performs the second optimization based on the optimal individual. This paper adopts the deletion point method to design the optimization operator (as shown in Figure 11). The deletion point method refers to the method of deleting redundant path points in the barrier-free continuous path so that the barrier-free continuous path becomes a barrier-free discontinuous path again. This barrier-free discontinuous path is a safe path with great performance indicators. The difference between the optimization operator and the previous deletion operator is that the deletion operator deletes the circular path in the unobstructed continuous path. After the deletion, it is still an unobstructed continuous path. But the optimization operator deletes the redundant path points in the unobstructed continuous path. The path after deletion is the barrier-free discontinuous path. Assuming that the path *path* contains n path points, *path(i)* represents the grid number corresponding to the *i*-th path point in the *path* path, and the optimization operator design steps are as follows:

(1) Initialize *i = 1*, *j = i + 1*, the *list* table is empty, add *path (i)* to the *list*.
(2) Determine whether j is equal to n. If they are equal, add *path (j)* to the *list* and go to step (4). If they are not equal, go to step (3).
(3) Determine whether the connection line between *i*-th path point and *j* + 1-th path points passes through the obstacle grid. If it does not pass, then *j = j + 1* and go to step (2). If it passes, then *i = j* and add *path (i)* to the *list*; then, go to step (3) again.
(4) Output the grid number in the *list*.

It is known from the optimization operator design steps. The grid number in the *list* table is the optimized result of the optimization operator.

## 5. Numerical Results and Analysis

### 5.1. Simulation Environment

This paper compares and analyzes the performance of MPMGA, SGA and PCDA algorithms in the natural simulation environment and the artificial simulation environment. Such as path generation, fitness and algorithm running time. The natural simulation environment refers to a simulation map that completely obeys the distribution of obstacles on the actual map. The natural simulation environment is mainly used to verify the feasibility and effectiveness of the algorithm, as well as various performance indicators, and enhance the application ability of the algorithm in real life. The artificial simulation environment refers to a simulation map that artificially sets the distribution of obstacles. The artificial simulation environment is mainly used to verify the adaptability of the algorithm in different environments. The software and hardware configuration of the simulation environment are shown in Table 1.

### 5.2. Natural Simulation Environment

**Figure 12:**
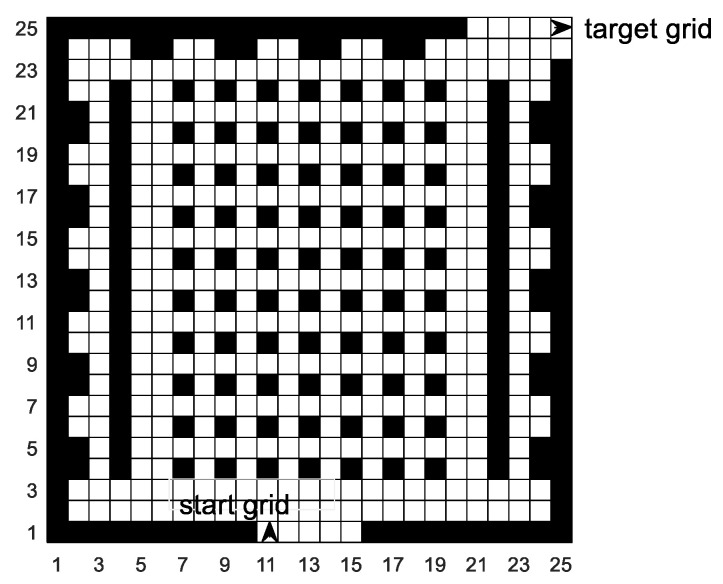
Simulation map environment.

The actual map environment simulated by the natural simulation environment is our school library. The library area is 30 m × 30 m. We divide the entire map into 25 × 25 grid models, and the actual area of each grid is 1.2 m × 1.2 m. According to the preprocessing process of grid model provided in Section 3, the 25 × 25 grid model is preprocessed, and the simulation map obtained is shown in Figure 12. In the simulation map, black obstacles represent unfeasible areas such as desks, sofas, bookcases, toilets, walls, and isolation belts and white grids represent feasible areas. The mobile robot enters from grid 10 and leaves from grid 624.

The parameter design of the three algorithms is shown in Table 2.

#### 5.2.1. Path Generation

**Figure 13:**
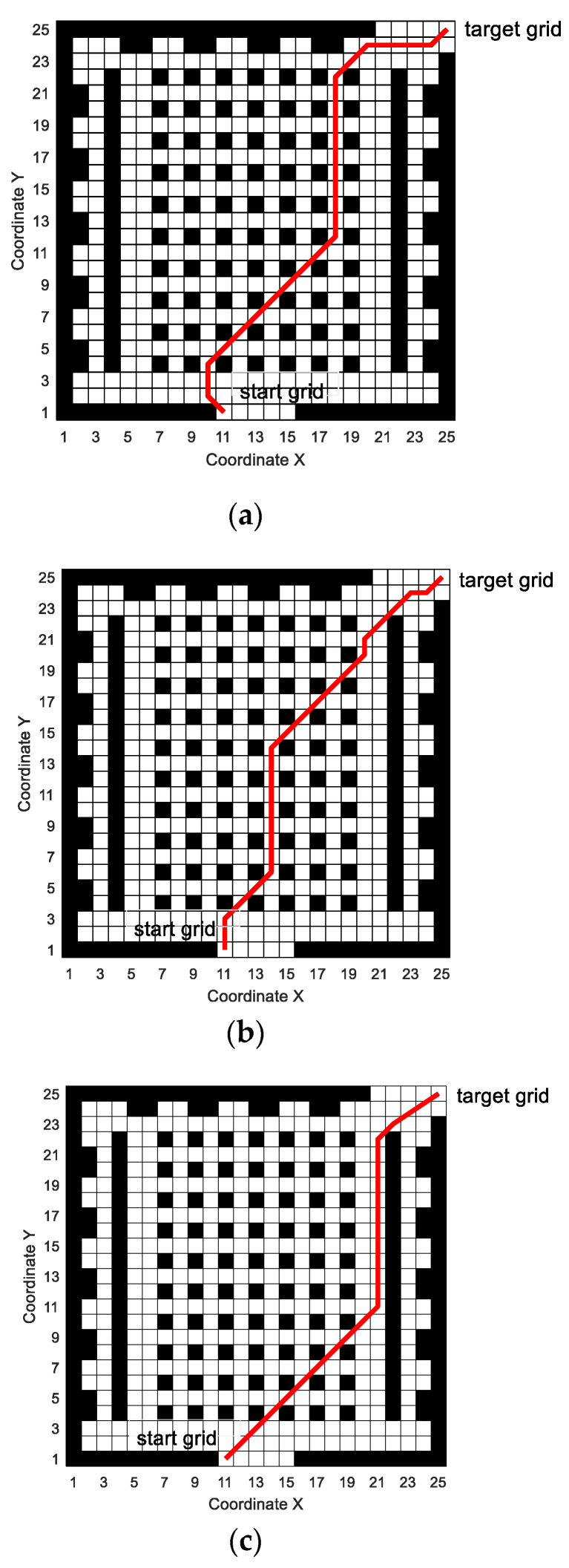
Simulated paths of the three algorithms. (**a**) SGA; (**b**) PCDA; (**c**) MPMGA.

The simulated paths of the three algorithms are shown in Figure 13. It can be seen from Figure 13 that all three algorithms can generate effective paths in the simulation map. MPMGA has the feasibility and effectiveness in the simulation map of the actual map and has the ability of practical application. In terms of the quality of the generated path, whether it is path length or path smoothness, the path generated by MPMGA is better than the path generated by PCDA and SGA.

**Figure 14:**
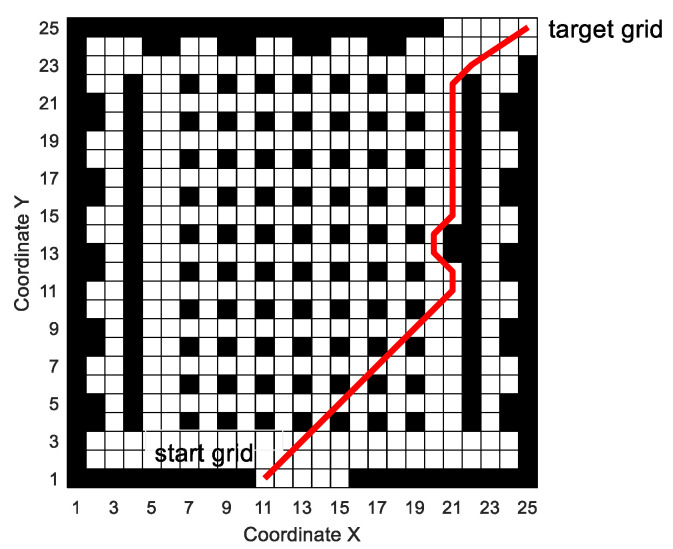
Mobile robot uses MPMGA for local path replanning.

Figure 14 shows the situation that the mobile robot encounters sudden obstacles and re-plans the local path when it moves according to the predetermined route. In Figure 14, the mobile robot moves according to the global path generated by MPMGA. In the process of driving according to the predetermined route, obstacles suddenly appear at the grids No. 320 and 345, blocking the path of the mobile robot. (here, two sudden obstacles are used to simulate two mobile pedestrians or mobile devices that suddenly appear on the predetermined path.)

When the mobile robot moves to the grid number 295, MPMGA is used to replan the local path. Taking the number 295 grid as the starting node, and the number 370 grid as the target node and performing local path planning, the path shown in Figure 14 is obtained. In the process of local path replanning, the time consumption is about 0.3~0.4 s. It can be seen that MPMGA can not only be used for global path planning to produce high-quality solutions, but also for local path re-planning to avoid sudden threats, and it can meet real-time requirements.

#### 5.2.2. Fitness Analysis

**Figure 15:**
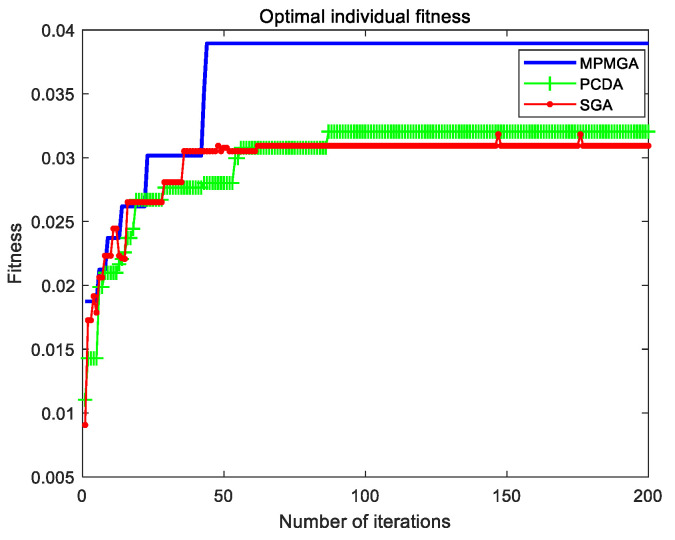
Evolutionary comparison diagram of optimal individual fitness.

The evolutionary comparison process of the optimal individual fitness of the three algorithms is shown in Figure 15. From the perspective of the quality of the first-generation optimal individuals, MPMGA is far superior to PCDA and SGA. This is mainly because MPMGA optimizes the quality of the initial population through connection operators and deletion operators. From the number of iterations of convergence, MPMGA converges around the 50th generation, PCDA converges around the 85th generation, and SGA converges around the 175th generation. MPMGA is superior to PCDA and SGA. Because MPMGA replaces the original selection operator with a migration operator. The migration operator can save high-quality individuals and further accelerate the convergence speed. From the perspective of the quality of convergent individuals, MPMGA is far superior to PCDA and SGA. This is because the optimization operator proposed by MPMGA can optimize the convergent individuals for the second time. Therefore, the optimization operator can greatly improve the quality of convergent individuals. 

From Figure 15, we can also find that SGA has a data drop phenomenon. The data drop phenomenon means that the optimal individual fitness of the next generation is worse than the optimal individual fitness of the previous generation. This phenomenon is caused by the traditional roulette selection strategy and the way of receiving cross-mutated individuals. Because MPMGA uses a migration operator and improves the receiving method of cross-mutated individuals, there is no data drop phenomenon in MPMGA.

**Figure 16:**
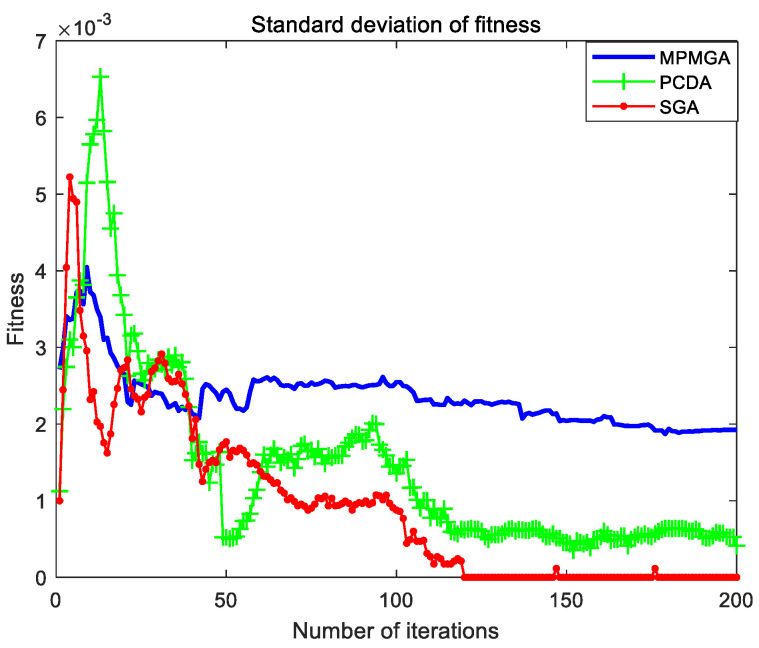
Evolutionary comparison diagram of population fitness standard deviation.

The evolutionary comparison process of population fitness standard deviation is shown in Figure 16. This paper uses the standard deviation of population fitness to measure the diversity of the entire population. It can be seen from Figure 16 that the standard deviation of population fitness produced by MPMGA has been maintained at a high level. This shows that the diversity of the entire population has been high during the evolution process, and the algorithm has been more active in exploring the entire solution space. The population fitness standard deviation produced by PCDA showed a short-term increase and then continued to decrease. This shows that the ability to explore the solution space of the algorithm is gradually enhanced at the beginning of the iteration. In the middle stage of the iteration, with the increase of the number of iterations, the ability of the algorithm to explore the solution space is gradually weakened. In the later stage of the iteration, the exploration ability of the algorithm has been maintained at a weak level. The standard deviation of population fitness produced by SGA also shows a trend of temporary increase and then a continuous decrease. After the 120th generation, the diversity of the entire population is zero, the phenomenon of population homogeneity is completely formed, the entire population is composed of the same individuals, and the crossover operator is completely invalid.

**Figure 17:**
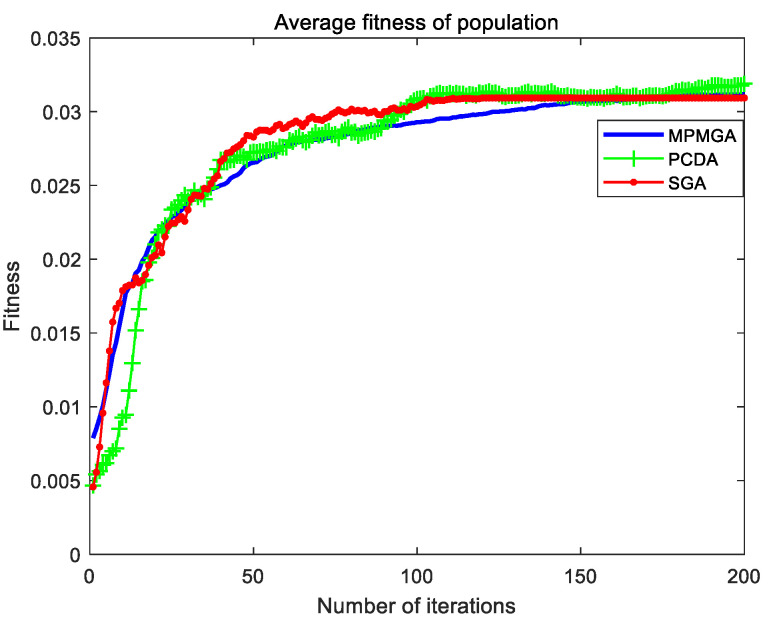
Evolutionary comparison diagram of population average fitness.

The evolutionary comparison process of population average fitness is shown in Figure 17. This paper uses the average fitness of the population to measure the evolution of the entire population. It can be seen from Figure 17 that the average fitness of the initial population produced by MPMGA is better than the average fitness of the initial population produced by PCDA and SGA. The main reason is that MPMGA improves the generation way of the initial population and produces many feasible high-quality first-generation individuals. However, from the perspective of the evolution of the entire population, the evolution curve produced by MPMGA is inferior to the evolution curve produced by PCDA and SGA. The evolution speed of the entire population is also slower. In fact, we need to use Figure 16 to analyze the evolution of the entire population. In Figure 16, SGA completely fell into homogeneity in the later stage of the iteration. The entire population is composed of the same individuals, and the population completely loses its evolutionary potential. Although PCDA did not completely fall into homogeneity in the later stage of the iteration, the diversity of the entire population was relatively poor. This means that the evolutionary potential of the entire population is weak. MPMGA has strong population diversity from beginning to end. This shows that the entire population has always had strong evolutionary potential. We can see from Figure 17 that although MPMGA evolves slowly, it has been evolving. In PCDA and SGA, the evolution of the entire population has stalled around the 105th generation. Therefore, through the two figures, we can analyze that PCDA and SGA evolve rapidly in the early stage of iteration, which is the illusion caused by homogenization. Essentially, a large number of identical individuals are produced in the entire population.

#### 5.2.3. Time Analysis

Table 3 shows the running time of each stage of MPMGA algorithm during the program running in detail. It can be seen from Table 3 that the program running time of MPMGA is longer than the program running time of PCDA and SGA. The main reason is that the initial population generation of MPMGA takes a relatively high time. This is mainly because MPMGA chooses to sacrifice a certain amount of time to improve the quality of the initial population. In the initial population generation, the connection operator and the deletion operator are introduced to optimize the initial population.

#### 5.2.4. Comprehensive Comparison

In the natural simulation environment, each algorithm is simulated 20 times, and then the average value is obtained (as shown in Table 4). 

It can be seen from Table 4 that MPMGA is better than PCDA and SGA in average fitness, average path length, average path smoothness, and average number of convergence iterations. But MPMGA is worse than PCDA and SGA in average program running time.

### 5.3. Artificial Simulation Environment

**Figure 18:**
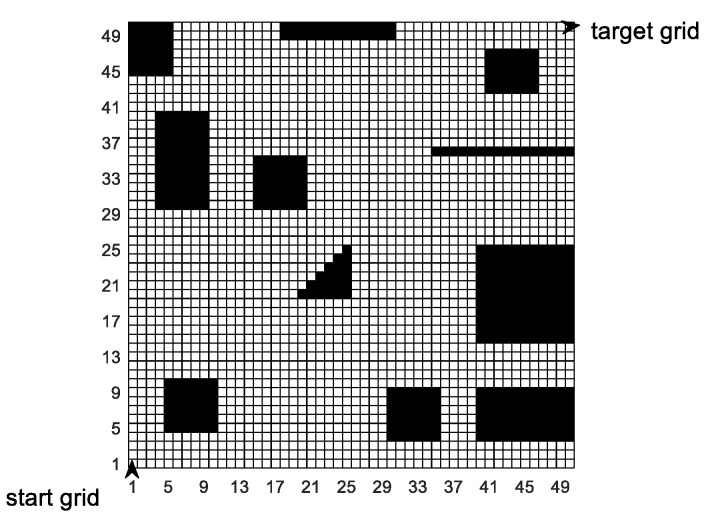
50 × 50 grid simulation map.

The second simulation environment is an artificial simulation environment, and the simulation map is a 50 × 50 grid model. The area of each grid is 1.2 m × 1.2 m, so the area of the simulation map is 60 m × 60 m. The simulation map is shown in Figure 18. In the simulation map, black obstacles represent infeasible areas, and white grids represent feasible areas. The mobile robot enters from grid 0 and leaves from grid 2499.

The parameter design of the three algorithms is shown in Table 5.

#### 5.3.1. Path Generation

**Figure 19:**
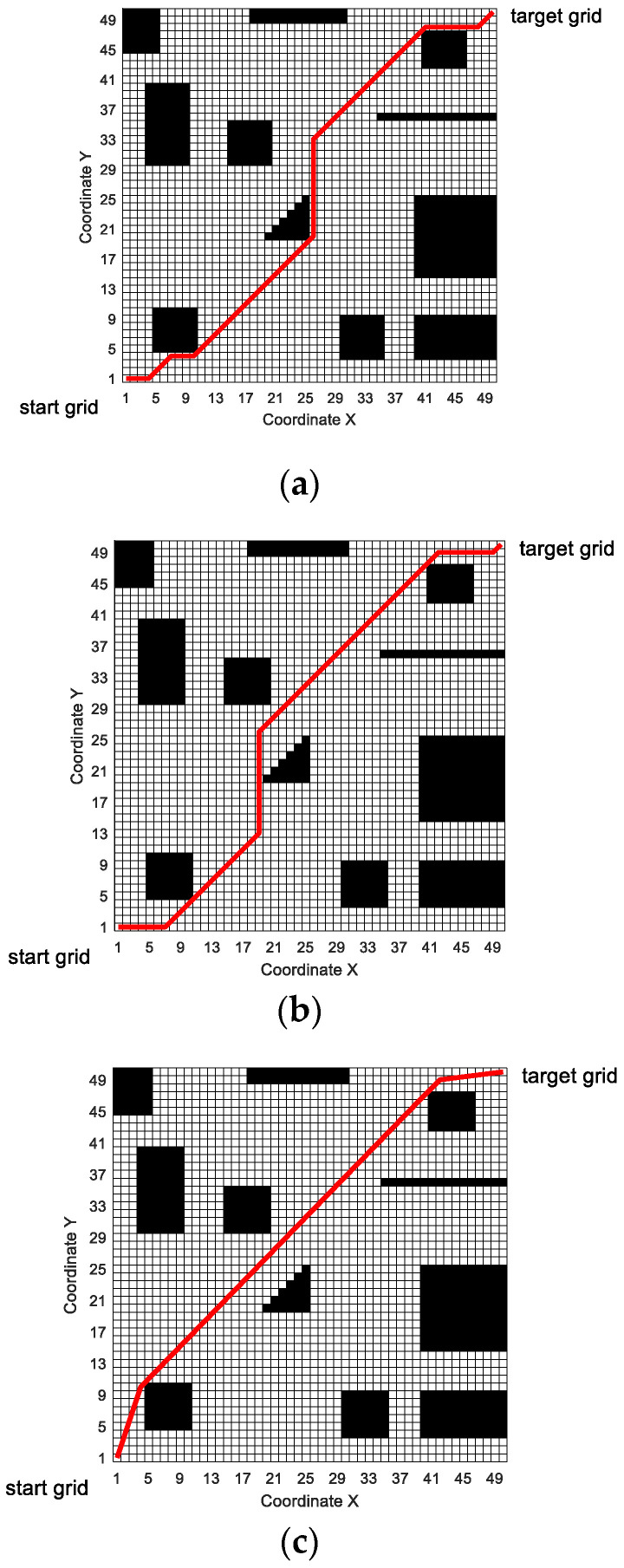
Simulated paths of the three algorithms. (**a**) SGA; (**b**) PCDA; (**c**) MPMGA.

The simulated paths of the three algorithms are shown in Figure 19. It can be seen from Figure 19 that MPMGA can still generate feasible and effective planning paths in a 50 × 50 grid simulation map. In terms of the quality of the generated path, whether it is path length or path smoothness, the path generated by MPMGA is better than the path generated by PCDA and SGA.

**Figure 20:**
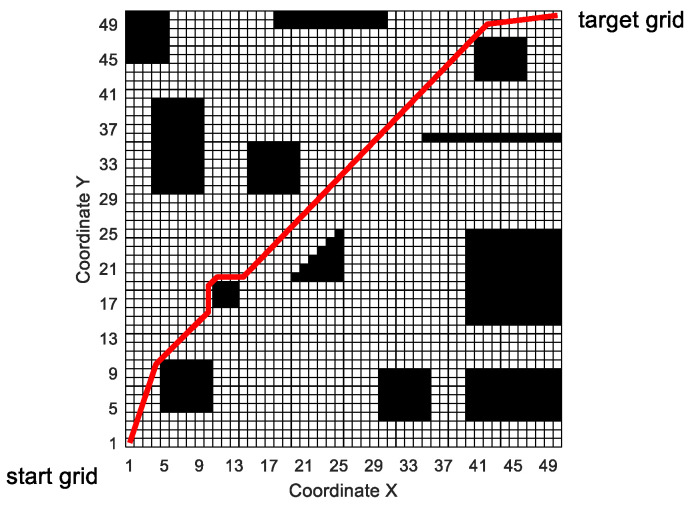
Mobile robot uses MPMGA for local path replanning.

Figure 20 shows the situation that the mobile robot encounters sudden obstacles and re-plans the local path when it moves according to the predetermined route. 

In Figure 20, the mobile robot moves according to the global path generated by MPMGA. In the process of driving according to the predetermined route, large obstacles suddenly appeared at nine grids numbered 810, 811, 812, 860, 861, 862, 910, 911, and 912, blocking the path of the mobile robot.

When the mobile robot moves to the grid number 759, MPMGA is used to replan the local path. Taking the number 759 grid as the starting node and the number 963 grid as the target node and performing local path planning, the path shown in Figure 20 is obtained. In the process of local path replanning, the time consumption is less than 1 s. Therefore, MPMGA meets the real-time requirement of local path planning in the artificial simulation environment.

#### 5.3.2. Fitness Analysis

**Figure 21:**
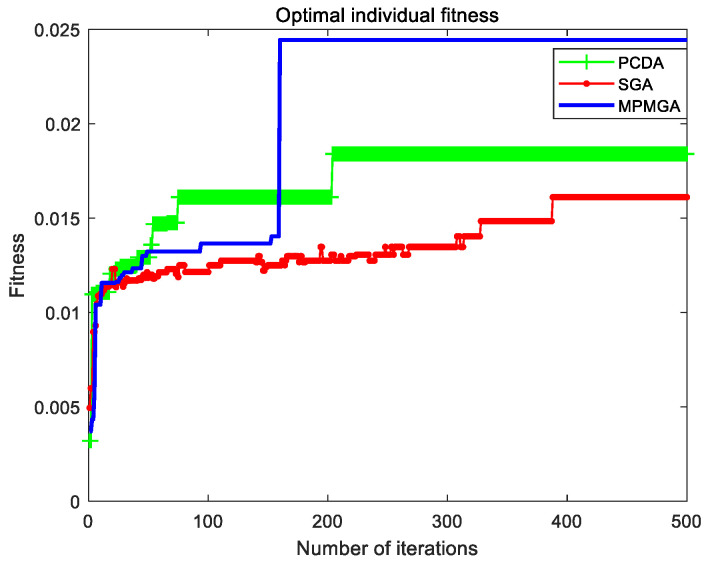
Evolutionary comparison diagram of optimal individual fitness.

**Figure 22:**
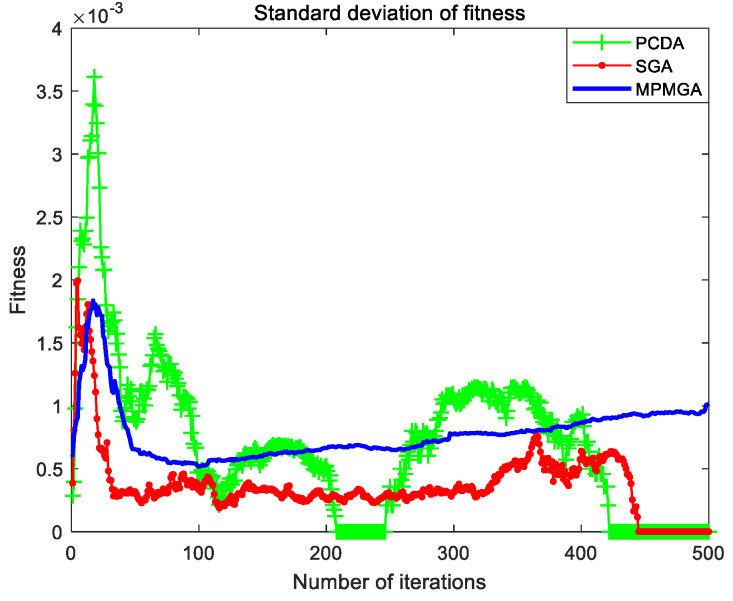
Evolutionary comparison diagram of population fitness standard deviation.

**Figure 23:**
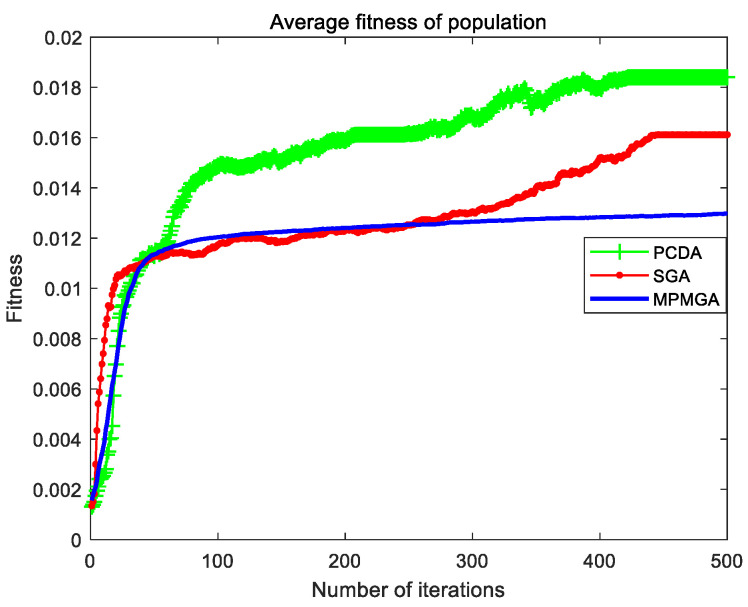
Evolutionary comparison diagram of population average fitness.

Figure 21 shows the evolutionary comparison process of optimal individual fitness under the current parameter settings. Figure 22 shows the evolutionary comparison process of the population fitness standard deviation under the current parameter settings. Figure 23 shows the evolutionary comparison process of the average fitness of the population under the current parameter settings.

The graph distributions shown in Figure 21, Figure 22 and Figure 23 are approximately the same as the graph distributions shown in Figure 15, Figure 16 and Figure 17. This indicates that no matter what kind of map environment, no matter how the obstacles are distributed, MPMGA is always better than PCDA and SGA.

#### 5.3.3. Time Analysis

Table 6 shows the running time of each stage of MPMGA algorithm during the program running in detail. Comparing Table 3 and Table 6, we can find that as the scale of the map increases, the proportion of the initial population generation time to the total time gradually increases. In the 50 × 50 grid map model, this proportion is as high as 80%. Therefore, for large-scale maps, it is necessary to reduce the initial population generation time and thus reduce the running time of the program.

#### 5.3.4. Comprehensive Comparison

In the artificial simulation environment, each algorithm is simulated 20 times, and then the average value is obtained (as shown in Table 7). It can be seen from Table 7 that MPMGA is better than PCDA and SGA in average fitness, average path length, average path smoothness, and average number of convergence iterations. But MPMGA is worse than PCDA and SGA in average program running time. 

The average program running time of MPMGA has reached an astonishing 120 s. Comparing Table 4 and Table 7, we can find that as the scale of the map increases, the program running time will also increase rapidly. From the above analysis, it can be concluded that:

(1) Whether in the natural simulation environment or the artificial simulation environment, MPMGA is feasible and effective. And MPMGA has the ability of application in the actual environment.
(2) No matter what scale of the map, no matter how the obstacles are distributed, the path planned by MPMGA is always better than the path planned by PCDA and SGA. In addition, MPMGA can make local path re-planning in the emergencies, so as to achieve emergency avoidance.
(3) MPMGA is better than PCDA and SGA in optimal individual fitness, average population fitness, standard deviation of population fitness, and optimal individual convergence iteration number. However, as the scale of the map increases, it takes a longer time for MPMGA to generate the initial population. In terms of the average program running time, MPMGA takes a longer time than PCDA and SGA.

## 6. Conclusions

In this paper, a multi-population migration genetic algorithm is proposed, and the framework and operators of the standard genetic algorithm are improved. In terms of framework, the algorithm proposes a parallel interactive framework. This framework has good parallelism and robustness. Especially when there are many individuals in the initial population, and the performance of a single processor is limited. A parallel interactive framework can divide a large population into several small populations, and each small population is equipped with a processor. The data among the small populations interacts through the bus, which can greatly reduce the running time of the program. Even if a processor fails and some data are lost, the impact on the algorithm is relatively limited. In terms of operators, the algorithm proposes a migration operator and an optimization operator. The migration operator replaces the selection system of the selection operator by the migration system, and the optimization operator performs the second optimization of the convergent optimal individual. In addition, the algorithm also improves the population’s initialization process, crossover operator, and mutation operator. By using the new operators or improving the original operators, the algorithm breaks the local optimal solution, solves the phenomenon of serious homogenization of population individuals, accelerates the convergence speed of the algorithm and improves the quality of convergent individuals. The simulation results show that MPMGA is not only suitable for simulation maps of various scales and various obstacle distributions, but also has superior performance. But the MPMGA program takes too long time to run.

In fact, if we consider implementing MPMGA on a real mobile robot, MPMGA may face the following problems: First, in actual large-scale scenarios, the MPMGA program takes a long time to run. Although MPMGA has excellent performance in large-scale maps, the long program running time limits the application of MPMGA in actual large-scale scenarios. Second, MPMGA is not sensitive to unknown environments. It means MPMGA does not consider how to generate a high-quality path in an unknown environment. We know that the map environment is unknown in many actual scenarios. In this case, the application of MPMGA has been greatly restricted. Third, MPMGA does not consider the preprocessing process of grid maps more comprehensively. This may cause MPMGA to fail to generate feasible paths in actual maps with a large number of irregular obstacles.

In the future, we need to do four aspects: First, we need to reduce the running time of the program. On the one hand, we can compress map models or simplify map models. On the other hand, we can improve the population’s initialization process. Second, we need to enhance the application capabilities of algorithms in different environments. MPMGA can perform static global path planning; it can also effectively deal with sudden threats and perform local path re-planning. However, the global or local path planning in an unknown environment is not considered. Therefore, global or local path planning in an unknown environment can also be used as the next research content. Third, we need to consider the preprocessing process of grid maps more comprehensively. We can use adaptive grid map method. In other words, the grid size in the grid map is no longer fixed. The size of the grid is automatically adjusted according to the size of the mobile robot and obstacles. The adaptive grid map method can effectively deal with the actual map with a large number of irregular obstacles. Fourth, we consider applying MPMGA on actual mobile robots. Mobile robots can obtain information about obstacles in the surrounding environment through visual sensors to generate electronic maps. We need to use the grid environment modeling method and map preprocessing process given in Section 3 to process the electronic map. After processing the electronic map, we get a grid map that algorithm can run. Then we can run the MPMGA program on the grid map to generate the actual path we need.

## Figures and Tables

**Table 1:** The software and hardware configuration.

**Hardware**	Processor	AMD Ryzen 5 4500U with Radeon Graphics 2.38 GHz
	RAM	8.00 GB (7.37 GB available)
**Software**	Operating System	Windows 10(64-bit operating system)
	Simulation Tool	Matlab r2018a

**Table 2:** Algorithm parameters.

**MPMGA**	Start grid number	10	
	Target grid number	624	
	Initial population size	60	
	The size of each small population	20	
	Number of iterations	200	
	Crossover probability	High crossover probability	0.8
		Low crossover probability	0.1
	Mutation probability	High mutation probability	0.8
		Low mutation probability	0.1
	Weight coefficient	α = 0.8, β = 0.2	
	Accuracy coefficient	1	
	The initial temperature	1	
	Temperature decay rate	0.7	
**PCDA/SGA**	Start grid number	10	
	Target grid number	624	
	Initial population size	60	
	Number of iterations	200	
	Crossover probability	0.8	
	Mutation probability	0.1	
	Weight coefficient	α = 0.8, β = 0.2	
	Accuracy coefficient	1	

**Table 3:** Comparison table of the running time of each segment program of the three algorithms.

Algorithm	Initial Population Generation Time (s)	Population Evolution Time (s)	Second Optimization Time (s)	Total Time (s)	The Proportion of the Initial Population Generation Time to the Total Time
MPMGA	1.4153	1.2616	0.136	2.8129	50.31%
PCDA	0.8494	1.1891	-	2.0385	41.67%
SGA	1.0065	1.2797	-	2.2862	44.03%

**Table 4:** Data comparison table of the three algorithms.

Algorithm	Average Fitness	Average Path Length (m)	Average Path Smoothness	Average Number of Convergence Iterations	Average Program Running Time (s)
MPMGA	0.03930	30.8085	15	69.1	2.7352
PCDA	0.03363	31.5877	24.09	87.6	1.9434
SGA	0.03053	32.5383	33.64	119.3	2.2678

**Table 5:** Algorithm parameters.

**MPMGA**	Start grid number	0	
	Target grid number	2499	
	Initial population size	120	
	The size of each small population	40	
	Number of iterations	500	
	Crossover probability	High crossover probability	0.8
		Low crossover probability	0.1
	Mutation probability	High mutation probability	0.8
		Low mutation probability	0.1
	Weight coefficient	α = 0.8, β = 0.2	
	Accuracy coefficient	1	
	The initial temperature	1	
	Temperature decay rate	0.7	
**PCDA/SGA**	Start grid number	0	
	Target grid number	2499	
	Initial population size	120	
	Number of iterations	500	
	Crossover probability	0.8	
	Mutation probability	0.1	
	Weight coefficient	α = 0.8, β = 0.2	
	Accuracy coefficient	1	

**Table 6:** Comparison table of the running time of each segment program of the three algorithms.

Algorithm	Initial Population Generation Time (s)	Population Evolution Time (s)	Second Optimization Time (s)	Total Time (s)	The Proportion of the Initial Population Generation Time to the Total Time
MPMGA	102.7476	18.8514	0.241	121.84	84.33%
PCDA	79.2217	20.1283	-	99.35	79.74%
SGA	84.7192	19.4608	-	104.18	81.32%

**Table 7:** Data comparison table of the three algorithms.

Algorithm	Average Fitness	Average Path Length (m)	Average Path Smoothness	Average Number of Convergence Iterations	Average Program Running Time (s)
MPMGA	0.02347	72.6975	20.5	179.8	120.5906
PCDA	0.01702	76.2673	34	207.3	96.5157
SGA	0.01554	76.9703	61.5	312.9	102.1386
